# Calcium Binding Protein S100A16 Expedites Proliferation, Invasion and Epithelial-Mesenchymal Transition Process in Gastric Cancer

**DOI:** 10.3389/fcell.2021.736929

**Published:** 2021-09-28

**Authors:** Xiaoying You, Min Li, Hongwei Cai, Wenwen Zhang, Ye Hong, Wenjie Gao, Yun Liu, Xiubin Liang, Tijun Wu, Fang Chen, Dongming Su

**Affiliations:** ^1^Department of Pathology, Nanjing Medical University, Nanjing, China; ^2^Department of Pathology, Women’s Hospital of Nanjing Medical University, Nanjing, China; ^3^Department of Clinical Laboratory, Children’s Hospital of Nanjing Medical University, Nanjing, China; ^4^Department of General Surgery, Second Affiliated Hospital, Nanjing Medical University, Nanjing, China; ^5^Department of Geratology, The First Affiliated Hospital of Nanjing Medical University, Nanjing, China; ^6^Department of Pathophysiology, Nanjing Medical University, Nanjing, China; ^7^Key Laboratory of Human Functional Genomics of Jiangsu Province, Nanjing Medical University, Nanjing, China; ^8^Department of Pathology and Clinical Laboratory, Sir Run Run Hospital, Nanjing Medical University, Nanjing, China

**Keywords:** S100A16, gastric cancer, metastasis, tight junctions, ZO-2

## Abstract

Gastric cancer (GC) is one of the most common malignant tumors of the digestive system, listed as the second cause of cancer-related deaths worldwide. S100 Calcium Binding Protein A16 (S100A16) is an acidic calcium-binding protein associated with several types of tumor progression. However, the function of S100A16 in GC is still not very clear. In this study, we analyzed S100A16 expression with the GEPIA database and the UALCAN cancer database. Meanwhile, 100 clinical GC samples were used for the evaluation of its role in the prognostic analysis. We found that S100A16 is significantly upregulated in GC tissues and closely correlated with poor prognosis in GC patients. Functional studies reveal that S100A16 overexpression triggers GC cell proliferation and migration both *in vivo* and *in vitro*; by contrast, S100A16 knockdown restricts the speed of GC cell growth and mobility. Proteomic analysis results reveal a large S100A16 interactome, which includes ZO-2 (Zonula Occludens-2), a master regulator of cell-to-cell tight junctions. Mechanistic assay results indicate that excessive S100A16 instigates GC cell invasion, migration, and epithelial-mesenchymal transition (EMT) via ZO-2 inhibition, which arose from S100A16-mediated ZO-2 ubiquitination and degradation. Our results not only reveal that S100A16 is a promising candidate biomarker in GC early diagnosis and prediction of metastasis, but also establish the therapeutic importance of targeting S100A16 to prevent ZO-2 loss and suppress GC metastasis and progression.

## Introduction

Gastric cancer (GC) is the fourth most commonly diagnosed cancer and the second leading cause of cancer-related deaths worldwide (Sitarz et al., 2018). Globally, an average of 989,600 new cases and 738,000 GC mortalities occur every year (Ferlay et al., 2010, 2015; Sitarz et al., 2018). Various risk factors are positively correlated with this fatal malignant tumor, including environmental factors, genetic instability, genetic factor defects, bacterial factors, i.e., *Helicobacter pylori* infection and host-related factors (Joshi and Badgwell, 2021). Although early GC screening improves overall survival, the prognosis of GC is still poor when compared with other solid tumors (Hamashima, 2014), since a majority of GC patients are still diagnosed with advanced stage, accompanied by regional, distant, or both, metastasis (Smyth et al., 2020). The treatment for GC patients is mainly radical surgical resection and chemoradiotherapy, while metastatic patients are less effective in surgical treatment, more resistant to drug therapy, and thus have lower survival rates (Peng et al., 2014; Zhao et al., 2015; Feng et al., 2019). Therefore, it is necessary to investigate the molecular mechanism underlying gastric carcinogenesis and progression, especially the key genes and signaling pathways that promote GC cells proliferation, invasion, and metastasis.

In humans, dysregulated expression patterns of the S100 proteins family is a common feature of tumor progression, of each type exhibits unique S100 protein profile or characteristics (Bresnick et al., 2015). S100 family is the largest subfamily of calcium (Ca^2+^)-binding proteins which are composed of 22 components that act as intracellular Ca^2+^ sensors and extracellular factors to regulate cellular responses (Yap et al., 1999; Zimmer and Weber, 2010). It has been widely indicated that S100 members participate in multiple stages of the tumorigenic process such as cell proliferation, tumor invasion, angiogenesis and immune evasion (Bresnick et al., 2015). Notably, S100 proteins interact with receptors for advanced glycation end-products (RAGE), p53 and p21, which play a role in the degradation of the extracellular matrix (ECM) and metastasis (Ji et al., 2014). S100 Calcium Binding Protein A16 (S100A16), the most recent member of S100 family proteins, functions to increase the tumor progression. The molecular mechanisms of S100A16 involving in the tumor metastasis are diverse in various malignant tumors, including pancreatic cancer (Fang et al., 2021; Zhuang et al., 2021), leukemia (Zhang et al., 2019), breast cancer (Zhou et al., 2014), and so on. In gastric cancer, decreased S100A16 expression is involved in *miR-6884-5p* and ADAMTS19 (A Disintegrin and Metalloproteinase with Thrombospondin motifs 19) inhibition of GC cells migration, invasion, and EMT (Lv et al., 2020; Jiang et al., 2021), however, the role of S100A16 itself in GC metastasis has not been fully clarified yet.

Tight junctions (TJs), as the topmost structure between epithelial cells and endothelial cells, are recognized as the control element for the diffusion of ions and certain molecules around the cell. It is becoming increasingly obvious that TJ plays a vital role in maintaining the integrity of cells, and the loss of cohesion of its structure can lead to the invasion and metastasis of cancer cells (Martin and Jiang, 2009; Bhat et al., 2018; Lauko et al., 2020). Zonula occludens proteins ZO-2 belongs to the membrane associated guanylate kinase homolog (MAGUK) protein family and is concentrated at the cytoplasmic face of TJs in epithelial cells (Umeda et al., 2006). Based on recent research, the interaction of ZO-2 with viral oncoproteins and kinases and its silencing in diverse carcinomas reinforce the view of ZO-2 as a tumor regulator protein (Yang et al., 2018; Akizuki et al., 2019; Gonzalez-Mariscal et al., 2019; Cong et al., 2020). In line with this, ZO-2 absence is also responsible for the diffusely infiltrating growth and frequent metastatic spread of GC cells (Kato et al., 2010; Shinto et al., 2010), while the specific molecular mechanism remains unclear.

In the current study, we demonstrated that S100A16 is considerably induced to trigger GC metastasis and progression via downregulating ZO-2 levels. We observed that S100A16 expression is dramatically increased in gastric cancer tissues compared with adjacent normal tissues, and S100A16 elevation is a significant prerequisite for GC proliferation, invasion, and migration. Using mass spectrometric analysis, we identified a large group of potential interactome for S100A16 in GC cells, including Zonula occludens proteins ZO-2 which acts as one core element in tight junctions. Moreover, we found that S100A16 is responsible for ZO-2 ubiquitination and degradation, resulting in the reduced cellular content of ZO-2, leading to the enhanced spread of GC metastasis.

## Materials and Methods

### Cell Culture and Treatment

The non-malignant human gastric mucosal epithelial cell line GES-1 (RRID: CVCL_EQ22) and human GC cell lines (MGC-803, RRID: CVCL_5334; SGC-7901, RRID: CVCL_0520) were purchased from ATCC. Cells were cultured in RPMI-1640 medium (Gibco, United States) with 10% fetal bovine serum (Wisent, China). The cells were cultured at 37°C in an incubator supplemented with 5% CO_2_.

### Gene Overexpression and Knockdown

Human lentivirus-S100A16, adenovirus-S100A16, lentivirus-ZO-2 and their control virus were purchased from Genechem (Shanghai, China). SGC-7901 and MGC-803 cell lines that stably overexpressing S100A16 and/or ZO-2 were established via being infected with lentivirus-S100A16 and/or ZO-2 as described previously (Fan et al., 2021). Specific siRNA for S100A16 (si-S100A16) was purchased from GenePharma (Shanghai, China). Transient transfection with interfering RNA (siRNA) and plasmids was performed with Lipofectamine 2000 (Invitrogen) according to manufacturer’s protocol.

### Patients and Tissue Specimens

The initial 8 cases of gastric cancer samples from patients were obtained as FFPE (formaldehyde-fixed and paraffin-embedded) tissues from the Department of Pathology in Sir Run-Run Hospital of Nanjing Medical University. The tissue microarray was purchased from the Shanghai Xinchao Biotechnology Co., Ltd. It contains 100 cases of gastric cancer and 80 cases of adjacent tissue sites, of which 80 cases are matched specimens.

### *In vivo* Tumor Formation

All procedures were approved by the Institutional Animal Care and Use Committee of Nanjing Medical University. 6-week-old male BALB/C nude mice were purchased from CAVENS LAB ANIMAL (China). For *in vivo* tumor formation, SGC-7901 cells, either stably overexpressing S100A16 or its control lentivirus, were resuspended in 1:1 PBS/Matrigel (356,237, Corning) solution and injected subcutaneously in the flank of nude mice at a density of 5 × 10^6^ cells in 0.1 ml per mouse. Mice were kept in a specific-pathogen-free (SPF) environment and tumor size was measured weekly since 5 days after injection. Four weeks after injection, euthanasia was administered, and tumors were isolated with the volume calculated according to the formula *Volume* = (*long diameter* × *short diameter*^2^)/2.

### Immunohistochemistry Staining

All protocols for animal experimentation and maintenance adhered to the guidelines of the Institutional Animal Care and Use Committee at Nanjing Medical University. IHC was performed as previously described (Fan et al., 2021). Briefly, tissues were deparaffinized and rehydrated firstly, then blocked with 5% BSA in PBS at room temperature for 30 min. Afterward, slides were incubated overnight at 4°C with antibodies against S100A16 (Sigma) and ZO-2 (Abcam) in blocking buffer. After being washed, the specimens were incubated in HRP-conjugated secondary antibody and visualized using a DAB Peroxidase Substrate Kit (Gene Tech). Images were obtained using a laser scanning microscope (IX-51, Olympus), and IHC scores were evaluated by three independent pathologists.

### Western Blotting and Immunoprecipitation

The total proteins of lysates from human GC cell lines (MGC-803 and SGC-7901) and GES-1 cell line were separated by 8 or 12% SDS-PAGE which containing 1.5 mol/l Tris-HCI (pH 8.8), 1 mol/l Tris-HCI (pH6.8), 30% Acr-Bis, 10% APS, 10% SDS and TEMED. 5% non-fat milk was used to block the unbound sites for 2 h at room temperature. The protein was blotted with primary antibodies against S100A16 (Proteintech Group), ZO-2 (Proteintech Group), E-Cadherin (Cell Signaling Technology), Twist (Cell Signaling Technology), Vimentin (Cell Signaling Technology) and then were incubated with secondary antibodies. GAPDH was detected as a loading control. For immunoprecipitation, whole cell proteins were extracted using NP40 lysis, the pull-down assay was performed as we described previously (Fan et al., 2021).

### RNA Isolation and Quantitative RT-PCR Analysis

Total RNA was extracted by TRIzol^TM^ Plus RNA Purification Kit (Invitrogen, United States) according to the manufacturer’s instructions. cDNA was prepared from RNA using the ReverTra Ace RT-PCR Kit (TOYOBO Biotech, Japan). All the RT-PCR reactions were performed with SYBR Green Master (Roche Molecular Systems, Switzerland). β-actin was used as an internal control.

### Cell Immunofluorescence Staining

For Immunofluorescence staining, the SGC-7901 cells and MGC-803 cells were fixed in 4% paraformaldehyde overnight at 4°C, followed by three washes with PBS carefully to remove any debris. After fixation, 0.2% Triton X-100 was used for cell membrane permeabilization. 15 min later, cells were blocked by 1% BSA at room temperature for 1 h and incubated overnight at 4°C with anti-S100A16 and anti-ZO-2 antibodies. The next day cells were washed with PBS for three times, then the cells were incubated with appropriate secondary antibodies for 30 min at 37°C. The cells were stained with 4,6-diamidino-2-phenylindole for 2 min at room temperature and then washed three times with PBS. All images were obtained from Olympus confocal microscope and processed using Photoshop software.

### Cell Proliferation and Colony Formation Assays

For the cell proliferation assay, 24 h after transfection, tumor cells were seeded into 96-well plates at a density of 1500 per well and cultured for 5 days. Each well was added with 100 μl 1% TCA for fixation overnight at 4°C daily. After being washed with ddH_2_O for 3-5 times, these plates were put into oven until no moisture remains. Each well was then added 50 μl Sulforhodamine B (SRB) for 30 min at room temperature and then washed with 1% acetic acid solution for 5 times, and this procedure was repeated. Finally, 100 μl 10 nmol/l Tris (pH = 10.5) was added into each plate to dissolve the SRB. The optical density at 490 nm (OD490), in linear correlation with the number of living cells, was measured by Varioskan^TM^ LUX microplate reader (Thermo Fisher Scientific). For the colony formation assay, tumor cells (500 cells per well) were seeded into 6-well plates. After 2 or 3 weeks of incubation, the cells were fixed in 4% paraformaldehyde and then stained with crystal violet for 30 min (Beyotime Biotechnology, China). The colonies were counted via Image J (version 1.8.0).

### Cell Migration Assays

Cell migration ability was measured using transwell chambers (8 μm pore size; Corning Costar, Cambridge, MA, United States). For the transwell assay, cells cultured in serum-free RPMI-1640 medium were seeded into the upper chamber. The lower chamber contained RPMI-1640 medium supplemented with 20% serum, which served as a chemoattractant. After 24 or 48 h incubation, the filters were fixed in 4% paraformaldehyde for 1h and stained with crystal violet. The upper faces of the filters were gently removed, and the lower faces with cells migrated across the filters were imaged and counted under the microscope (IX-51, Olympus).

### Cell Invasion Assays

The Millicell^TM^ hanging cell culture inserts (MCMP24, Millipore, United States) were coated with Matrigel^TM^ Matrix (354, 234, Corning, United States) as described in the manufacturer’s protocol. After 24 h of serum-starvation, MGC-803 or SGC-7901 cells (5 × 10^4^) were plated in the pre-coated insert in 200 μl serum-free medium and incubated in a 24-well plate containing 0.75 ml medium with 10% FBS per well for 24 h. With non-invasive cells on the upper surface of the membrane removed with a cotton swab, invasive cells that migrated through the membrane and adhered to the lower surface were fixed with methanol and stained with crystal violet staining solution (Beyotime Biotechnology). The number of invasive cells per field was quantified using an inverted microscope imaging system (IX-51, Olympus).

### Mass Spectrometry Analysis

The mass spectrometry was performed on the precipitated protein of SGC-7901 cells transfected with Lentivirus-S100A16 or empty vectors. The labeled peptides were analyzed on the LTQ-Orbitrap instrument (Thermo Fisher Scientific, United States) connecting to a Nano ACQUITY UPLC system via a nanospray source. The LC-MS/MS was operated in positive ion mode. The analytical condition was set at a linear gradient from 0 to 60% of buffer B (CH3CN) in 150 min, and flow rate of 200 nl/min. For analysis of proteins from SGC-7901 cells, one full MS/MS scan was followed by five MS/MS scans on those five highest peaks respectively. The MS/MS spectra acquired from precursor ions were submitted to Maxquant (version 1.2.2.5) using the following search parameters: the database searched was Uniprot proteome (version20120418); the enzyme was trypsin (KR/P); the dynamic modifications were set for oxidized Met (+ 16); carbamidomethylation of cysteine was set as static modification; MS/MS tolerance was set at 10ppm; the minimum peptide length was 6; the false detection rate for peptides, proteins were all set below 0.01 (Wu et al., 2020). Mass spectrometry data that support the findings of this study have been deposited in the ProteomeXchange Datasets under accession code PXD028022.

### Wound Closure Assay

Cells were plated in 3.5-cm dishes and were divided into 12-well plates to achieve 70-80% confluence and then wounded by dragging a plastic pipette tip across the monolayer surface three times, followed by gentle washing with PBS twice. Then cells were grown in serum-free culture medium for 24 h until the digital images of cells migrated into the scratch were taken on an inverted microscope. Images of the wounds were recorded with a Leica DM IRB inverted microscope (Solms, Germany).

### Measurement of MMP-2, MMP-9, and TIMP-1 Levels

The supernatants of SGC-7901 and MGC-803 cells were collected after indicated treatment, and the concentrations of matrix metalloproteinase-2 (MMP-2), MMP-9, and tissue inhibitor of metalloproteinase-1 (TIMP-1) were measured by ELISA kit (USCN Life Science, China) according to the manufacturer’s instructions.

### Cycloheximide Chase Assay

SGC-7901 cells infected with lenti-S100A16 or lenti-scramble were seeded in 6-well plates at a density of 1 × 10^5^ cells per well and cultured overnight. Then, cells were treated with 50 μg/ml of cycloheximide (CHX) and harvested after 0/12/24/48h of CHX treatment. The total protein was extracted and subjected to SDS-PAGE to detect the protein level of ZO-2 at different time points, indicating ZO-2 stability in SGC-7901 cells overexpressing S100A16.

### Plasmid Construction

hZO-2-Flag, hZO-2ΔU2 region-Flag, hS100A16-Myc were generated using a QuikChange Site-Directed Mutagenesis kit (Stratagene, La Jolla, CA, United States) according to manufacturer’s instructions.

### Cell-Based Ubiquitination Assay

HEK293A cells were transfected with plasmids encoding hZO-2-Flag or hZO-2ΔU2 region-Flag, hS100A16-Myc, and Ubiquitin-HA. After transfection for 24 h, the cells were incubated with the proteasome inhibitor MG132 (Sigma Aldrich) for 4 h and then lysed. Equal amounts of total cell lysates were incubated with the Flag antibodies (Sigma) overnight at 2°C. Immunocomplexes were collected overnight at 4°C using protein-A sepharose beads (Roche). The immunoprecipitates were washed with lysis buffer and subjected to Western blot analysis with anti-HA antibody (Cell Signaling Technology).

### Statistical Analysis

GraphPad Prism 8.0.1 (GraphPad Software, Inc., La Jolla, CA, United States) and Microsoft Excel 2019 were used for statistical analysis and the data were presented as mean ± standard deviation. Data were statistically analyzed using one-way ANOVA with a Bonferroni correction, followed by Fisher’s exact test for comparison of two groups.

## Results

### S100A16 Is Elevated in Gastric Cancer Tissues and Correlated With Poor Prognosis in Gastric Cancer Patients

**FIGURE 1:**
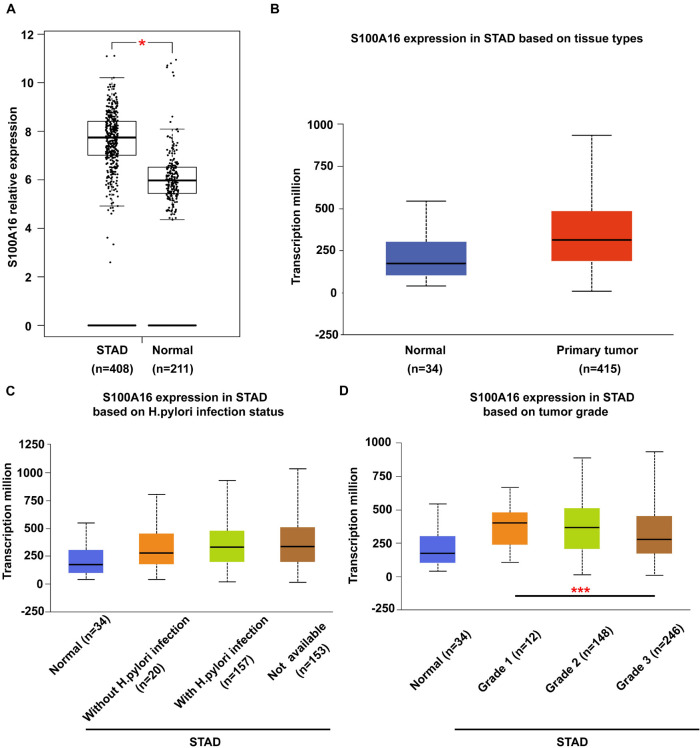
Bioinformatic analysis of S100A16 expression in GC. **(A,B)** S100A16 expression in human GC tissues according to GEPIA and UALCAN database. **(C)** Boxplot showing expression of S100A16 associated with *H. pylori* infection status. **(D)** Boxplot showing relative expression of S100A16 in normal individuals or GC patients with grade 1, 2, or 3 tumors. For panels **(A–D)**, *P < 0.05 and ***P < 0.001 vs. normal group or Grade 1 STAD.

It has been widely reported before that S100A16 is upregulated in various types of cancer cells in the comparison with the normal tissues. To further determine the role of S100A16 in the progression of GC, we analyzed S100A16 expression in GC tissues with GEPIA^1^ and UALCAN^2^ databases. S100A16 was significantly more excessive in GC tissues when compared with normal tissues (Figures 1A,B). Based on this, we utilized UALCAN database to explore clinic pathological features of S100A16 in GC samples. As shown in Figures 1C,D, S100A16 expression was positively correlated with H. pylori infection (a recognized GC pathogenic factor), as well as with the tumor grade of GC. These findings indicated that S100A16 could serve as a valuable diagnostic and prognostic indicator in GC.

**FIGURE 2:**
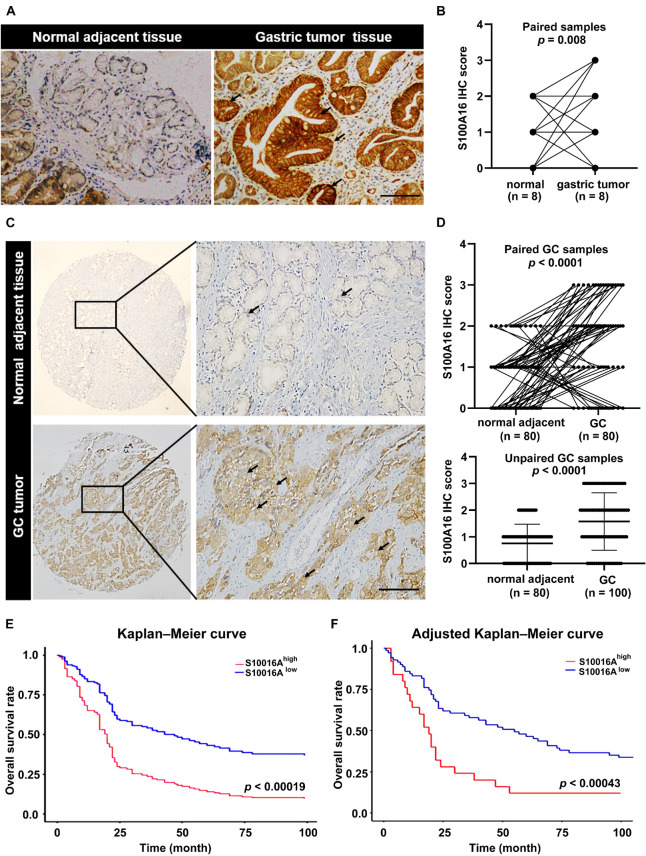
S100A16 is upregulated in GC tissues and correlated with poor prognosis in GC patients. **(A)** IHC staining of S100A16 in gastric tumors compared with normal adjacent tissues obtained from the Department of Pathology in Sir Run-Run Hospital of Nanjing Medical University. Scare bar = 200 μm. **(B)** Quantification of immunohistochemical staining in paired gastric samples (*n* = 8). **(C)** IHC staining of S100A16 in GC tissues compared with normal adjacent tissues, both of which obtained from purchased tissue microarray. Scare bar = 200 μm. **(D)** Paired line plots showing S100A16 IHC score in matched gastric cancer tissues (*n* = 80, upper). Unpaired line plots showing S100A16 IHC score in gastric cancer tissues [normal adjacent tissues (*n* = 80) GC tissues (*n* = 100), lower]. **(E)** Kaplan–Meier survival analysis of S100A16^high^ and S100A16^low^ GC patients mentioned in panel **(C)**. **(F)** Adjusted Kaplan-Meier curves of overall survival rate in panel **(E)**.

We then confirmed the database analysis mentioned above. Using gastroscopic biopsy tissues obtained from GC patients, we demonstrated that S100A16 expression in GC tissues was dramatically higher than that in adjacent normal tissues (Figures 2A,B). Similarly, elevated S100A16 was also observed in GC tumors when compared with adjacent normal tissues according to tissue microarray (patient clinicopathological characteristics shown in Table 1), whether it was matched or not (Figures 2C,D). We further studied the relationship between S100A16 and clinical GC pathology. According to the IHC score for S100A16 (Table 2), all GC patients were divided into 2 groups: S100A16high (IHC score ≥ 2, 25 cases) and S100A16low (IHC score < 2, 72 cases). Kaplan-Meier analysis revealed that S100A16high patients had a worse prognosis than those in low expression groups (Figure 2E). The results above were still statistically significant (Figure 2F, *p* < 0.00043) after adjustment for other factors (such as gender, pathological grade, tumor size, and Ki67 index, shown in Table 3). Therefore, both bioinformatics and clinicopathological analysis prove that S100A16 is highly expressed in GC, which plays a role in GC progression.

**TABLE 1:** Clinicopathological characteristics GC patients in purchased microarray.

Characteristics	Cases
**Clinical information**	
Median age years [(range)]	65 (32-81)
Gender (male/female)	(64/36)
**Characteristics of tumor**	
Median volume of tumor [cm(range)]	5 (1.2-20)
Pathological grade (II/III/IV)	13/76/11
Vascular invasion (Yes/No)	14/86
Distant metastasis (Yes/No)	8/92
**Pathological features**	
Differentiation (high/media/low)	15/74/11
Median survival time [month(range)]	24 (1-107)
Survival cases	28
Death cases	69
Loss of follow-up	3

**TABLE 2:** Distribution of each factor for S100A16A^high^ and S100A16^low^ groups.

	low expression (*N* = 72)	high expression (*N* = 25)	Statistics	*P*-value
**Gender**				
Male	49	13	χ^2^ = 2.31	0.126
Female	23	12		
**Pathological grade**				
II	7	4	ω = 1022.5	0.29
III	56	20		
IV	9	1		
Tumor size(cm)	5.83(3.64)^a^	6.55(3.32)	*t* = −0.91	0.37
Ki67	0.16(0.21)	0.23(0.29)	ω = 640.5	0.032

*^a^Average (standard deviation).*

**TABLE 3:** Multivariate Cox regression fitting results.

Variable	Estimated effect	HR	95%CI	*P*-value
Expression	0.986	2.681	1.545∼4.643	0.00043
Gender	–0.107	0.899	0.535∼1.510	0.686
Pathological grade	0.649	1.914	1.095∼3.345	0.0228
Tumor size	0.079	1.082	1.011∼1.159	0.0239
Ki67	–0.389	0.678	0.252∼1.824	0.4415

**FIGURE 3:**
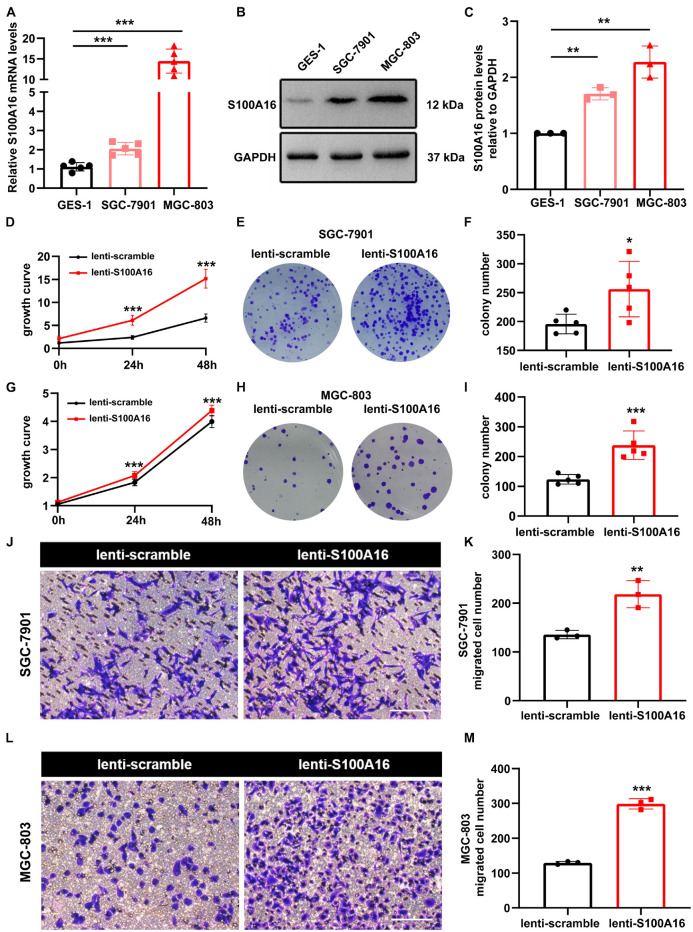
Overexpression of S100A16 accelerates GC cells growth and migration *in vitro*. **(A,B)** The mRNA and protein levels of S100A16 in GES-1, SGC-7901, and MGC-803 cells. GAPDH was used as internal standard. **(C)** Gray density of panel **(B)**. **(D)** Cell growth was measured by SRB staining in SGC-7901 cells stably overexpressing S100A16. **(E)** Colonies formed by SGC-7901 cells that stably overexpressing S100A16. **(F)** Colonies number in € were quantified. **(G)** Cell growth was measured by SRB staining in MGC-803 cells stably overexpressing S100A16. **(H)** Colonies formed by MGC-803 cells that stably overexpressing S100A16. **(I)** Colonies number in panel **(H)** were quantified. **(J,K)** The migration ability of SGC-7901 cells stably overexpressing S100A16 was measured by transwell assay. Migrated cell numbers were counted in panel **(K)**. Scare bar = 100 μm. **(L,M)** The migration ability of MGC-803 cells stably overexpressing S100A16 was measured by transwell assay. Migrated cell numbers were counted in panel **(M)**. Scare bar = 100 μm. Data are presented as mean ± SD. *N* = 3-15 for each group. For panels **(A,C)**, *P < 0.05, **P < 0.01, and ***P < 0.001 vs. GES-1 cells. For panels **(D**,**F**,**G**,**I**,**K**,**M)**, *P < 0.05, **P < 0.01, and ***P < 0.001 vs. lenti-scramble group.

**FIGURE 4:**
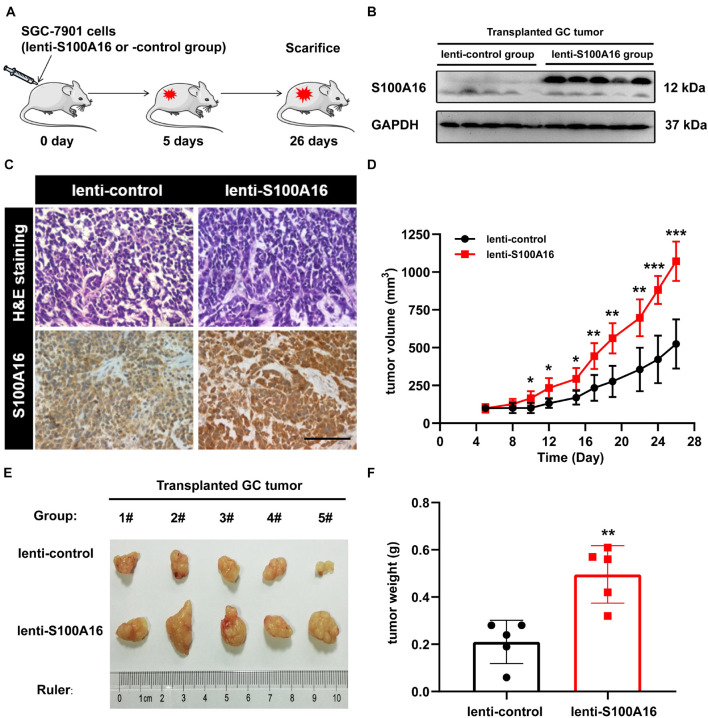
Overexpression of S100A16 enhances GC cells tumorigenesis *in vivo*. **(A)** Diagram of subcutaneous tumor transplantation in nude mice. **(B,C)** S100A16 overexpression were confirmed by Western blotting **(B)** and immunohistochemistry **(C)** in transplanted tumor tissues formed by SGC-7901 cells. Scare bar = 200 μm. **(D)** The volume for transplanted GC tumor was measured every 4-5 days since 5 d after injection. **(E,F)** The size and weight for xenograft tumors after sacrificed. Data are presented as mean ± SD. *N* = 5 for each group. For panels **(D,F)**, *P < 0.05, **P < 0.01, and ***P < 0.001 vs. lenti-control group.

In consistent with IHC results in Figure 2, we observed elevated S100A16 mRNA and protein levels in human GC cell lines MGC-803 and SGC-7901 when compared with gastric mucosa cell line GES-1 (Figures 3A–C). We next applied those two cell lines to investigate the biological function of S100A16 in GC cells. As shown in Figure 3D and Supplementary Figure 1A, S100A16 stably overexpression promoted the growth rate of SGC-7901. Colony formation assays further indicated that S100A16 overexpression enhances the capacity of SGC-7901 proliferation (Figures 3E,F). The pro-proliferation effect of S100A16 was also observed in MGC-803 cells (Figures 3G–I and Supplementary Figure 1B). Besides, migration capacities were assessed to be notably increased in SGC-7901 and MGC-803 overexpressing S100A16 by transwell assays (Figures 3J–M). To further evaluate the effect of S100A16 on GC cell migration and invasion *in vivo*, we constructed subcutaneous tumor formation experiments in nude mice using SGC-7901 cells stably infected with lenti-S100A16 or scramble control (lenti-S10016 or control group, Figure 4A). S100A16 overexpression in transplanted GC tumor tissues was confirmed in Figures 4B,C via Western blotting and IHC. The growth rate of tumor grafts in the mice from lenti-S100A16 group was significantly faster than that in the control-group from the 10th day of SGC-7901 infection (Figure 4D). We then sacrificed those mice and extracted the transplanted tumor tissues for observation. As we expected, the size and weight of tumor grafts isolated from lenti-S100A16 group were obviously larger than that in the control group after 26 days of tumor implantation (Figures 4E,F).

Collectively, S100A16 promotes GC cells migration, proliferation, and growth.

### S100A16 Inhibition Suppresses Gastric Cancer Cells Growth and Migration

**FIGURE 5:**
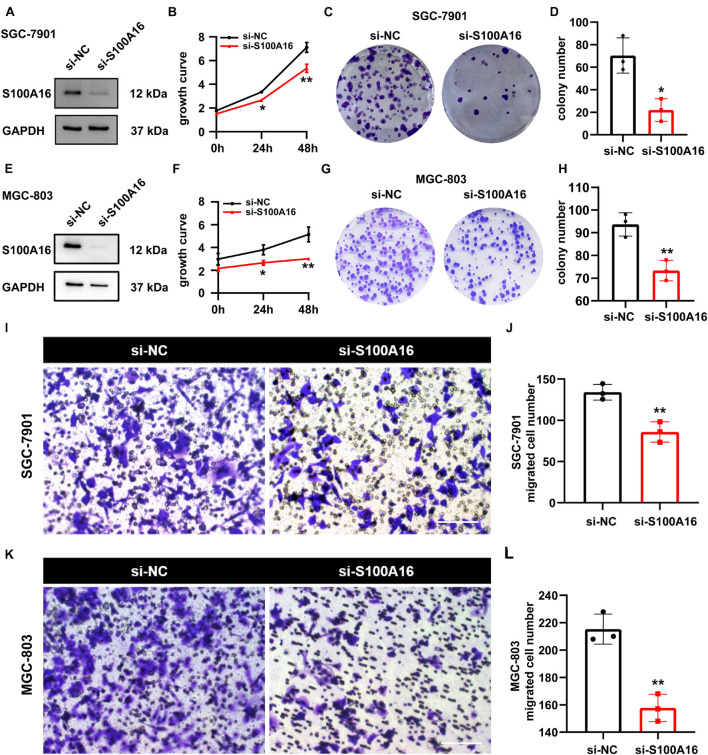
S100A16 inhibition suppresses GC cells growth and migration. **(A)** S100A16 knockdown in SGC-7901 cells were confirmed by Western Blotting. **(B)** Cell growth was measured by SRB staining in SGC-7901 cells transfected with si-NC and si-S100A16. **(C)** Colonies formed by SGC-7901 cells in panel **(B)**. **(D)** Colonies number in panel **(C)** were quantified. **(E)** S100A16 knockdown in MGC-803 cells were confirmed by Western Blotting. **(F)** Cell growth was measured by SRB staining in MGC-803 cells transfected with si-NC and si-S100A16. **(G)** Colonies formed by MGC-803 cells in panel **(F)**. **(H)** Colonies number in panel **(G)** were quantified. **(I,J)** The migration ability of SGC-7901 cells transfected with si-NC and si-S100A16 was measured by transwell assay. Migrated cell numbers were counted in panel **(J)**. Scare bar = 100 μm. **(K,L)** The migration ability of MGC-803 cells transfected with si-NC and si-S100A16 was measured by transwell assay. Migrated cell numbers were counted in panel **(L)**. Scare bar = 100 μm. Data are presented as mean ± SD. *N* = 3 for each group. For panels **(B,D,F,H,J,L)**, *P < 0.05 and **P < 0.01 vs. si-NC group.

We applied loss-of-function experiments to further determine the role of S100A16 in GC cells proliferation and migration. As shown in Figures 5A–D, S100A16 knock-down restricted the growth rate and colony formation of SGC-7901 cells. Also, similar results were verified in MGC-803 cells (Figures 5E–H). Furthermore, transwell assay results also demonstrated that S100A16 knockdown suppressed the migration of MGC-803 and SGC-7901 cells (Figures 5I–L). Together, S100A16 upregulation is both necessary and sufficient to trigger GC tumor growth, proliferation, and migration, which suggest that it functions as a considerable oncogene in GC.

### S100A16 and ZO-2 Interact Directly and Their Expression Levels Are Inversely Correlated in Gastric Cancer Cells

**FIGURE 6:**
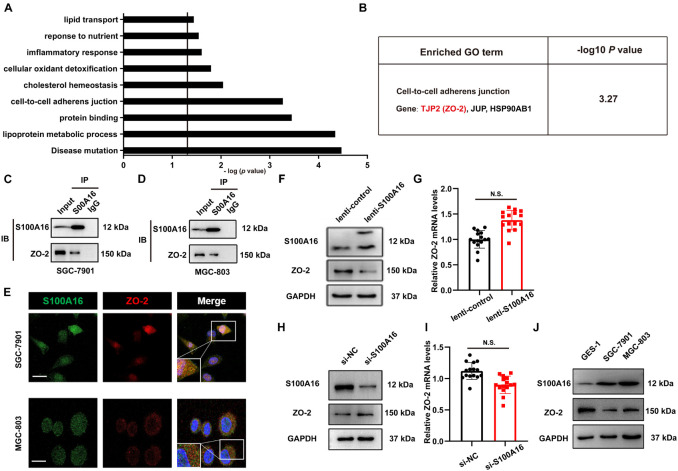
S100A16 and ZO-2 interact directly and their expression levels are inversely correlated in GC cells. **(A)** DAVID Bioinformatics Resources 6.7 was used to perform GO analysis of S100A16-binding proteins in SGC-7901 cells transfected with lenti-S100A16 or lenti-control (A). Relative cellular processes were sorted by −log (*P*-value). *P*-value < 0.05; −log (*P*-value) > 1.3. **(B)** A cluster of genes (ZO-2, JUP, HSP90AB1) associated with the cell-to cell adherens junction **(B)** was sorted out. **(C,D)** Validation of the interactions between endogenous S100A16 and ZO-2 in SGC-7901 **(C)** and MGC-803 cells **(D)**. **(E)** ZO-2 co-localizes with S100A16 in gastric cancer cells. Immunofluorescence staining was performed for ZO-2 (red), S100A16 (green), and DAPI (blue). Scale bars = 10 μm. **(F)** The whole cell protein levels of S100A16 in SGC-7901 cells stably overexpressing S100A16 were measured through Western blot analysis. **(G)** *ZO-2* mRNA level in SGC-7901 cells in panel **(F)** was detected via qRT-PCR assay. **(H)** The whole cell protein levels of S100A16 in SGC-7901 cells transfected with si-negative control (NC) or si-S100A16 for 60 h were measured through Western blot analysis. **(I)** *ZO-2* mRNA level in SGC-7901 cells in panel **(H)** was detected via qRT-PCR assay. **(J)** The expression of S100A16 and ZO-2 in GES-1, SGC-7901, and MGC-803 cells.

To determine the molecular basis of the increased proliferation and migration in GC cells with high S100A16 expression, we performed a mass spectrometric analysis to investigate the binding proteins of S100A16 in lenti-S100A16- or lenti-scramble-infected SGC-7901 cells. Data showed that 67 proteins interacted with S100A16, among which 25 proteins showed weakened (8 proteins) or stronger (17 proteins) affinity with S100A16 when S100A16 was highly expressed in SGC-7901 cells (Supplementary Table 1). Importantly, GO analysis with DAVID Bioinformatics Resources 6.7 (Figure 6A) showed that 3 of those 25 S100A16-interacting proteins were involved in cell-to-cell adherens junction which may be associated with tumor cell migration and invasion. Among these proteins is TJP2 (also called ZO-2), which is an important regulator in tumor growth and metastasis (Figure 6B and Supplementary Figure 2A). Immunoprecipitation analyses confirmed that endogenously S100A16 and ZO-2 coexisted in the precipitated complexes obtained from SGC-7901 cells (Figure 6C), which was also observed in MGC-803 cells (Figure 6D). Consistently, immunofluorescence staining results revealed that S100A16 co-localized with ZO-2 in both two GC cell lines (Figure 6E). Using SGC-7901 cells, we determined the inverse correlation between ZO-2 and S100A16 in GC cells. S100A16 overexpression significantly reduced ZO-2 expression at the translational rather than transcriptional level (Figures 6F,G and Supplementary Figure 2B); by contrast, immunoblot results revealed the slight but obvious accumulation of ZO-2 in S100A16 knockdown SGC-7901 cells, although its mRNA levels did not significantly change (Figures 6H,I and Supplementary Figure 2C). Moreover, we observed that ZO-2 protein levels were obviously declined in GC cells compared with normal gastric mucosal cell lines (GES-1), which was inversely correlated with S100A16 expression (Figure 6J and Supplementary Figure 2D).

### S100A16-ZO-2 Axis Mediates Migration, Invasion and Epithelial-Mesenchymal Transition in GC Cells

**FIGURE 7:**
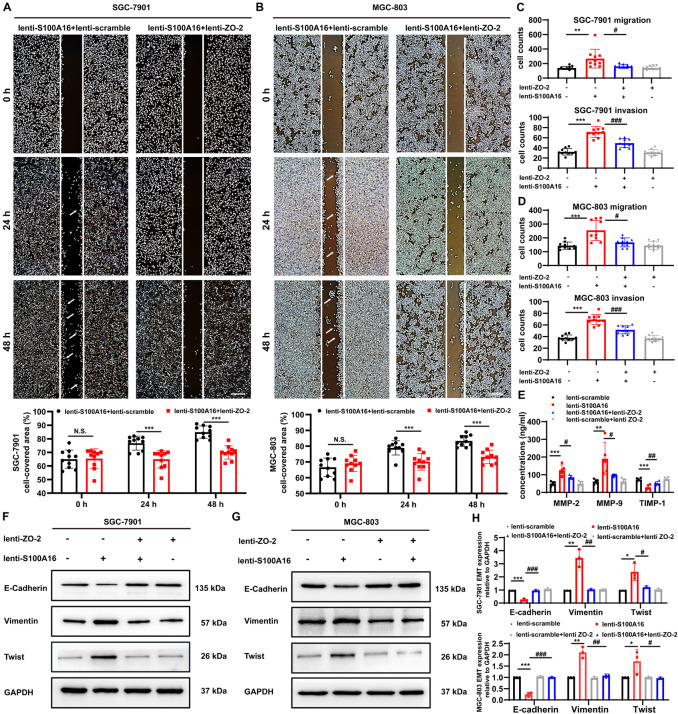
S100A16 triggers the process of invasion, migration and EMT in GC cells via ZO-2 inhibition. **(A,B)** The scratch wound-healing assays of SGC-7901 **(A)** and MGC-803 cells **(B)** stably overexpressing S100A16/ZO-2 or singly S100A16. Quantitative analysis was done below. Scare bar = 100 μm. **(C,D)** SGC-7901 **(C)** and MGC-803 cells **(D)** migration and invasion were measured by transwell assays. Cells were quantified. **(E)** MMP-2, 9 and TIMP-1 levels in culture supernatant of SGC-7901 cells were measured by ELISA assays. **(F–H)** The protein levels of EMT markers (E-Cadherin, Vimentin and Twist) in SGC-7901 **(F)** and MGC-803 cells **(G)**. GAPDH was used as internal standard. Gray density was quantified in panel **(H)**. Data are presented as mean ± SD. *N* = 3-10 for each group. For panels **(A,B)**, *P < 0.05, **P < 0.01, and ***P < 0.001 vs. lenti-S100A16 + lenti-scramble group. For panels **(C–E,H)**, *P < 0.05, **P < 0.01, and ***P < 0.001 vs. lenti-scramble group, ^#^*P* < 0.05, ^##^*P* < 0.01, and ^###^*P* < 0.001 vs. lenti-S100A16 group.

It has been indicated that the loss of ZO-2 participates in the processes involving epithelial-mesenchymal transition (EMT), diffusely infiltrating growth, and ultimately frequent metastatic spread of GC cells (Shinto et al., 2010), which is consistent with our observations that declined ZO-2 levels were correlated with poor survival rate in GC patients (Supplementary Figures 3A–C). To measure the role of ZO-2 in S100A16-induced GC cells metastasis, we performed several additional experiments. Results from the wound healing assay indicated that the migratory speed was significantly faster in SGC-7901 cells and MGC cells stably overexpressing lenti-S100A16 alone, but this enhancement could be reversed by ZO-2 replenishment (Figures 7A,B). Similarly, we applied transwell assays and found that both migration and invasion ability was promoted by elevated S100A16 levels, which could also be reverted by ZO-2 overexpression (Figures 7C,D). Moreover, compared with lenti-scramble group, the concentrations of matrix metalloproteinases-2, 9 (MMP-2 and 9) in SGC-7901 stably overexpressing S100A16 culture supernatant obviously rose while tissue inhibitor of metalloproteinase-1 (TIMP-1) dramatically reduced, which was partially reversed by ZO-2 supplement (Figure 7E), suggesting that S100A16-medicated ZO-2 inhibition contributes to GC invasion and metastasis. Notably, it has been widely recognized that the process of EMT facilitates tumor cells proliferation, motility, and invasion during tumor development, including gastric tumorigenesis and progression (Christiansen and Rajasekaran, 2006; Natalwala et al., 2008; Peng et al., 2014) we next analyzed the protein levels of EMT markers including epithelial marker E-Cadherin, mesenchymal marker Vimentin as well as EMT promoting transcription factor Twist. As shown in Figures 7F–H, S100A16 overexpression alone downregulated E-Cadherin levels but upregulated Vimentin and Twist levels, which was barely seen in the S100A16-ZO-2 overexpression group. Together, ZO-2 inhibition participates in the effects of S100A16 on invasion, migration and EMT phenotype of gastric cancer cells.

### The Cellular Content of ZO-2 Diminishes After S100A16 Overexpression Due to Ubiquitin-Proteasome Mediated Degradation

**FIGURE 8:**
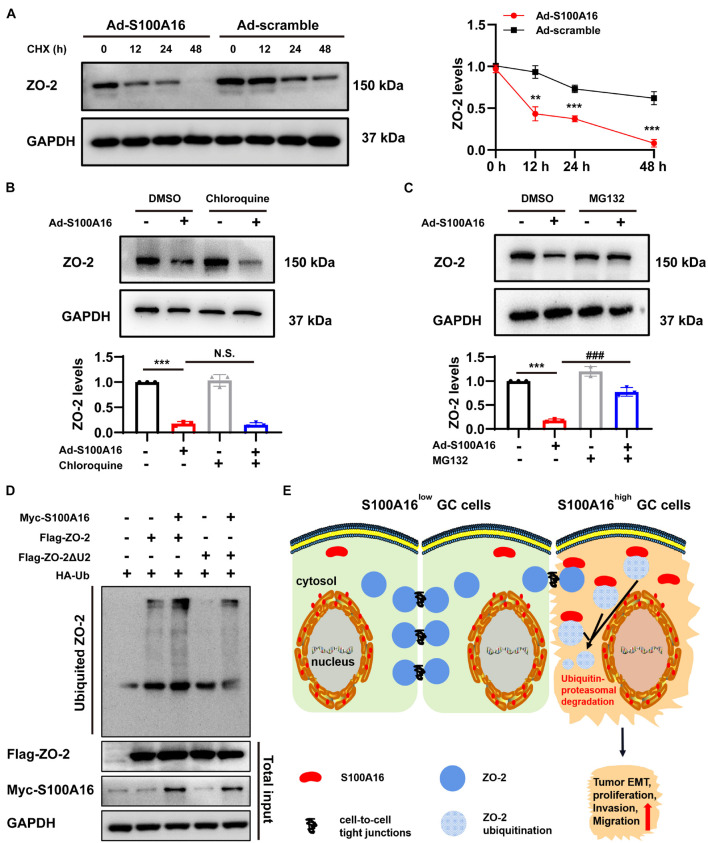
S100A16 contributes to ZO-2 ubiquitination and degradation. **(A)** SGC-7901 cells were transfected with Ad-S100A16 or Ad-control for 6 h. After co-culture with 50 mmol/l cycloheximide (CHX) for 0, 12, 24, or 48 h, Western blot analysis was performed, and the relative ZO-2 expression was calculated on the right. **(B)** SGC-7901 cells were transfected with Ad-control or Ad-S100A16 for 6 h and then treated with or without chloroquine (50 μmol/l) for another 48 h; ZO-2 expression was measured through Western blot analysis. **(C)** SGC-7901 cells were transfected with Ad-control or Ad-S100A16 for 6 h and then treated with or without MG132 (10 μmol/l) for another 48 h; ZO-2 expression was measured through Western blot analysis. **(D)** SGC-7901 cells were co-transfected with Ub-HA, S100A16-Myc, and ZO-2-Flag or ZO-2-ΔU2 region-Flag expression plasmids for 18 h; 6 h after treatment with proteasome inhibitor MG132 (10 μmol/l), the cells were lysed, and the supernatant was collected for an IP experiment. Flag-Tag antibody was used for immunoprecipitation, and HA-Tag antibody was used for Western blot analysis to measure the level of exogenous ZO-2 ubiquitination. **(E)** A proposed model of S100A16 mediating ZO-2 inhibition via ubiquitin-proteasomal degradation in GC metastasis. Data are presented as mean ± SD. *N* = 3 for each group. For panels **(A–C)**, **P < 0.01, and ***P < 0.001 vs. Ad-scramble group, ^###^*P* < 0.001 vs. Ad-S100A16 group.

We then investigated the mechanism underlying S100A16-mediated ZO-2 inhibition. Given that S100A16 did not disturb ZO-2 mRNA levels in GC cells (Figures 6G,I), we speculated that excessive S100A16 may weaken the stability of ZO-2 proteins. As expected, S100A16 overexpression accelerated the decay of ZO-2 proteins (Figure 8A). To figure out whether decreased ZO-2 was owning to proteasomal or lysosomal degradation, we applied cycloheximide (CHX) chase assays with MG132 (proteasomal degradation inhibitor) or chloroquine (lysosomal degradation inhibitor). As the data shown in Figures 8B,C, it was MG132 but not chloroquine that blocks the decline of ZO-2 protein levels induced by S100A16 overexpression. Consistently, S100A16 mediated the obvious increase in ZO-2 ubiquitination (Figure 8D). Interestingly, the lack of the Unique 2 (U2) region in ZO-2 diminished the pronounced ubiquitination caused by S100A16 (Figure 8D). Collectively, these results clearly demonstrate that S100A16 is responsible for ZO-2 ubiquitylation and degradation.

## Discussion

Gastric cancer (GC) is one of the most common malignant tumors worldwide, causing a tremendous threat to human health. Globally, about 70% of gastric cancer cases occur in developing countries, especially in China. GC has been the fourth leading cause of cancer-related death, meaning nearly 7.39% of patients diagnosed with gastric cancer died from the disease every year (Wu et al., 2021). The occurrence of gastric cancer is associated with multiple factors, including genetic factors, environmental factors. family history, diet, alcohol consumption, smoking, and *Helicobacter pylori* infection. Distant metastasis of tumors is one of the reasons for the high recurrence and mortality rate of gastric cancer patients.

In this study, we explored the role of S100A16, a Ca^2+^-binding protein, in GC progression and metastasis. Analysis of databases and clinical samples reveals that S100A16^high^ is positively correlated with poor prognosis and low overall survival rate of GC. Excessive S100A16 expression in GC cells leads to active proliferation and migration, whereas S100A16 inhibition effectively weakens the ability of growth and invasion in GC cells. When highly expressed, the interaction between S100A16 and ZO-2 (which acts as a core element in cell-to-cell tight junctions) was strengthened, and then contributed to the ubiquitination and degradation of ZO-2, weakening its biological function and eventually leading to elevated GC cells invasion and migration (Figure 8E).

The S100 protein family, which belongs to the superfamily of Ca^2+^-binding proteins, has been reported involving the progression of various cancers. S100A4 protein is one of the most extensively studied S100 family members, which is now considered as a valuable biomarker for cancer diagnosis and metastasis prediction (Kim et al., 2008; Wang et al., 2010; Tang et al., 2017). By contrast, expression of S100A8/S100A9 in sera has been reported to be associated with recurrence-free survival with bladder cancer (Minami et al., 2010). According to previous studies, S100A16 is upregulated in bladder cancer, lung cancer, pancreatic cancer, colorectal cancer and ovary cancer (Zhou et al., 2014; Bai et al., 2018; Sun et al., 2018; Fang et al., 2021; Zhuang et al., 2021), but the role of S100A16 in GC has not been fully elucidated before. Data from current study demonstrated that S100A16 expression was significantly elevated in GC tissues. Moreover, GC cells with higher S100A16 expression tended to have a robust capacity for proliferation, migration and invasion. *In vivo* and *in vitro* analyses revealed that ectopic overexpression of S100A16 promotes tumor formation and migration, whereas S100A16 knockdown remarkably reduced tumor growth and migration. All of these observations reveal that S100A16 upregulation is both necessary and sufficient to trigger GC cells proliferation and invasion, ultimately leading to GC metastasis. However, the mechanism underlying S100A16 elevation in GC tumors is still unclear and remains to be investigated.

In the current studies, we applied mass spectrometry to figure out the molecular mechanisms underlying the pathological roles of S100A16 in GC. We investigated for the first time the interactive proteins of S100A16 in GC cells, and we found that tight junction-associated molecule Zona occludens-2 (ZO-2, also called TJP2) bind directly with S100A16, and the interaction between those two proteins became stronger when S100A16 elevated. We observed that ZO-2 and S100A16 expression levels were inversely correlated in GC cells. In addition, ZO-2 replenishment significantly alleviated the accelerated GC cells invasion, migration and EMT caused by excessive S100A16.

ZO-2, a cytoplasmic protein of tight junctions, is a master regulator of gene expression, cell proliferation, cytoarchitecture, and cell size (Gonzalez-Mariscal et al., 2019). Studies have shown that ZO-2 expression is decreased in breast cancer, lung cancer, and other tumors (Martin et al., 2004; Paschoud et al., 2007; Gao et al., 2018). It has been noticed that ZO-2 downregulation reduces the protective function of tight junctions between cells and is related to tumor metastasis (Luczka et al., 2013; Akizuki et al., 2019), suggesting that ZO-2 may function as a tumor suppressor protein. Other studies have demonstrated that ZO-2 inhibition is involved in the diffusely infiltrating growth and frequent metastatic spread of GC cells (Kato et al., 2010; Shinto et al., 2010). Our research further clarifies the reason for declined ZO-2 in GC tumors, especially in tumor tissues with active proliferation and invasion, meaning S100A16 induces ZO-2 ubiquitination which is responsible for the abnormal ZO-2 degradation via ubiquitin-proteasomal system (UPS). In fact, ZO-2 could be degraded via both the ubiquitin-proteasomal and the lysosomal-mediated pathway, which depends on intracellular Ca^2+^ concentrations (Amaya et al., 2019). Interestingly, although S100A16 is a well-known Ca^2+^-binding protein, we observed that it binds with ZO-2 and mediated its degradation via UPS but not the lysosomal pathway. UPS is one of the main mechanisms of cellular protein degradation. The degraded proteins are labeled through ubiquitination and then degraded by 26S proteasomes. To combine ubiquitin with proteins, three distinct enzymes are needed: E1 (ubiquitin-activating enzyme), E2 (ubiquitin-conjugating enzyme) and E3 (ubiquitin ligase); E3 ubiquitin ligases recognize certain protein substrates and catalyzes the transfer of activated ubiquitin to client proteins. The targeting specificity of proteins degraded by ubiquitin proteasomes is mediated by E3 ubiquitin ligases, which contain a large family of members (Buetow and Huang, 2016; Senft et al., 2018; Bernassola et al., 2019; Fujita et al., 2019). Obviously, no studies have shown that S100A16 could serve as one E3 ligase for targeting proteins to ubiquitinated. Therefore, we speculate that S100A16 may act as a scaffold or bridge protein between ZO-2 and certain E3 ligase (s) that specifically recognize ZO-2. Back to our results, S100A16-induced ZO-2 ubiquitination was dramatically ameliorated when the unique 2 region (U2) of ZO-2 was deleted. Notably, the U2 region of ZO-2 was found critical for the interaction with 14-3-3 proteins which protect ZO-2 from degradation via proteasomes (Amaya et al., 2019). Thus, we assumed that S100A16 recruits some E3 ligase (s) to bind with ZO-2 via its U2 region, and then forms a complex that leads to ZO-2 degradation.

## Conclusion

In summary, our observations demonstrate that excessive S100A16-mediated ZO-2 ubiquitination and degradation play a crucial role in the progression of GC. This identification suggests that the therapeutic targeting of S100A16 could increase ZO-2 protein level to inhibit tumor invasion and metastasis for the treatment of GC.

## Data Availability Statement

The original contributions presented in the study are publicly available. This data can be found here: http://proteomecentral.proteomexchange.org/cgi/GetDataset, PXD028022.

## Ethics Statement

The studies involving human participants were reviewed and approved by Ethics Committee and Institutional Animal Care and Use Committee of Nanjing Medical University. The patients/participants provided their written informed consent to participate in this study. The animal study was reviewed and approved by the Institutional Animal Care and Use Committee of Nanjing Medical University. Written informed consent was obtained from the individual(s) for the publication of any potentially identifiable images or data included in this article.

## Author Contributions

DS and FC did the conceptualization, wrote, reviewed, and edited the manuscript, and supervised the data. XY, TW, WZ, ML, and HC performed the methodology. DS, FC, and XY carried out the formal analysis. XY, TW, YH, WG, and YL investigated the data. XL and DS carried out the resources and funding acquisition. TW wrote the original draft. XY visualized the data. All authors reviewed and commended on the manuscript.

## Conflict of Interest

The authors declare that the research was conducted in the absence of any commercial or financial relationships that could be construed as a potential conflict of interest.

## Publisher’s Note

All claims expressed in this article are solely those of the authors and do not necessarily represent those of their affiliated organizations, or those of the publisher, the editors and the reviewers. Any product that may be evaluated in this article, or claim that may be made by its manufacturer, is not guaranteed or endorsed by the publisher.

## Footnotes

1
http://gepia.cancer-pku.cn/

2
http://ualcan.path.uab.edu

## Abbreviations

GC: Gastric cancer
S100A16: S100 Calcium Binding Protein A16
EMT: epithelial-to-mesenchymal transition
ZO-2: Zonula Occludens-2
TJs: Tight junctions.
