# Supplementary material for: Calcium Binding Protein S100A16 Expedites Proliferation, Invasion and Epithelial-Mesenchymal Transition Process in Gastric Cancer
Source: Front Cell Dev Biol. 2021 Sep 28;9:736929. doi: 10.3389/fcell.2021.736929 (PMC8505768; doi:10.3389/fcell.2021.736929)
Supplement: Supplementary file 1 [file Data_Sheet_1.docx]

**SUPPLEMENTARY INFORMATION**

**Supplementary Figure S1:**

**
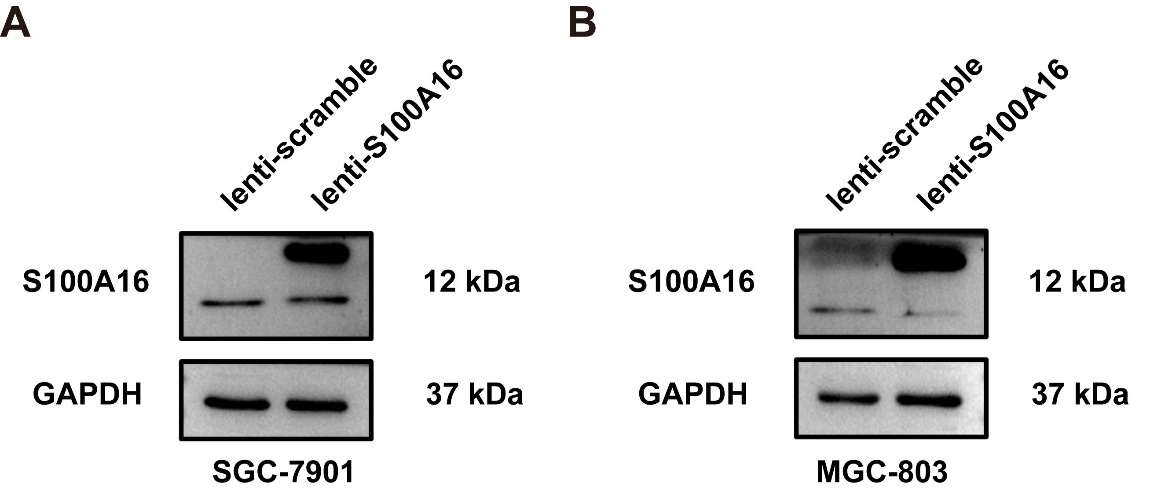
**

**Figure S1.** (**A**) S100A16 overexpression in SGC-7901 cells were confirmed by Western Blotting. (**B**) S100A16 overexpression in MGC-803 cells were confirmed by Western Blotting.

**Supplementary Figure S2:**

**
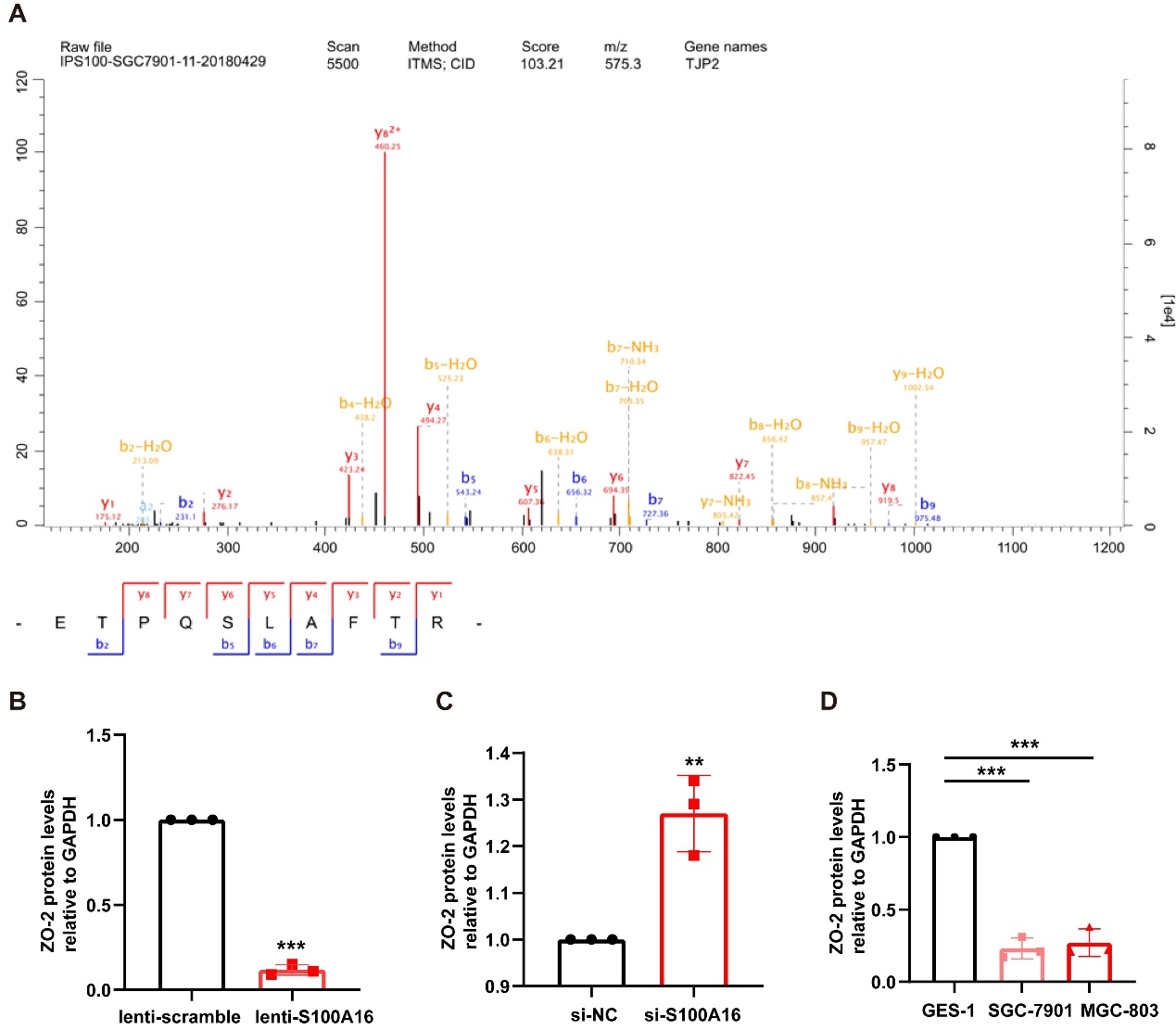
**

**Figure S2.** (**A**) A digested peptide from ZO-2 in LC-MS/MS analysis of protein complex coimmunoprecipitated with S100A16 in SGC-7901 cells. (**B**) Grey density of Figure 6E. (**C**) Grey density of Figure 6H. (**D**) Grey density of Figure 6J. Data are presented as mean ± SD. n = 3 for each group. For **B-D**, * *P* < 0.05, ** *P* < 0.01, *** *P* < 0.001 vs. lenti-scramble group or si-NC group or GES-1.

**Supplementary Figure S3:**


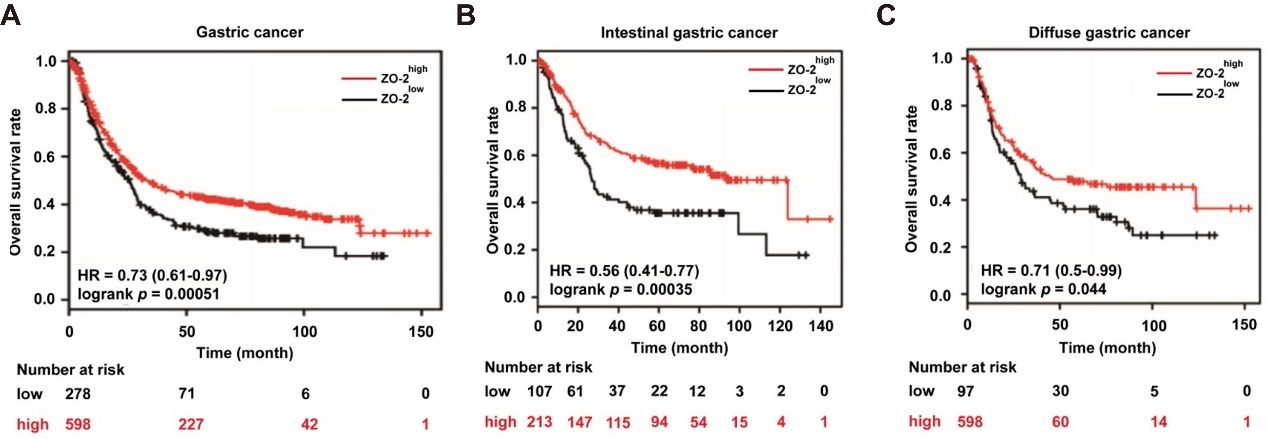


**Figure S3.** (**A-C**) Kaplan–Meier survival analysis of ZO-2^high^ and ZO-2^low^ gastric cancer patients.

**Supplementary Table S1.** List of the interactive proteins of S100A16 in SGC-7901 cells analyzed by mass spectrometry.

| **Gene** | **Protein** | **^a^NC** | **^b^S100A16** | **b/a ratio** |
| --- | --- | --- | --- | --- |
| PRSS1 | Trypsin-1 (Fragment) | + | - |  |
| MTFR1 | Mitochondrial fission regulator 1 (Fragment) | + | - |  |
| ZNF280A | Suppressor of hairy wing homolog 1 (Drosophila) | + | - |  |
| HIST1H4A | Histone H4 | + | - |  |
| KAT5 | Histone acetyltransferase KAT5 (Fragment) | + | - |  |
| CARD18 | Caspase recruitment domain-containing protein 18 | + | - |  |
| ERF | ETS domain-containing transcription factor ERF(Fragment) | + | - |  |
| EEF1A1P5 | Putative elongation factor 1-alpha-like 3 | + | - |  |
| PELO | Protein pelota homolog | + | - |  |
| SERPINA1 | Alpha-1-antitrypsin (Fragment) | + | - |  |
| PMEPA1 | Isoform 3 of Protein TMEPAI | + | - |  |
| DNAH10 | Dynein heavy chain 10, axonemal | + | - |  |
| TNRC6A | Trinucleotide repeat-containing gene 6A protein (Fragment) | + | - |  |
| MAGEB3 | Melanoma-associated antigen B3 | + | - |  |
| ZNF184 | Zinc finger protein 184 | + | - |  |
| SLC8A3 | Isoform 8 of Sodium/calcium exchanger 3 | + | - |  |
| HIST1H2BN | Histone H2B | + | - |  |
| DSG1 | Desmoglein-1 | + | - |  |
| POLE2 | Isoform 2 of DNA polymerase epsilon subunit 2 | + | - |  |
| CARMIL1 | F-actin-uncapping protein LRRC16A (Fragment) | + | - |  |
| FLNA | Filamin-A | + | - |  |
| AIM1 | Absent in melanoma 1 protein | + | - |  |
| CNTRL | Isoform 3 of Centriolin | + | + | 0 |
| TJP2 | Tight junction protein ZO-2 | + | + | 0.01 |
| DNAH3 | Dynein heavy chain 3, axonemal | + | + | 0.04 |
| ROS1 | Tyrosine-protein kinase receptor | + | + | 0.15 |
| JUP | Junction plakoglobin | + | + | 0.2 |
| S100A8 | Protein S100-A8 | + | + | 0.44 |
| HSP90AB1 | Heat shock protein HSP 90-beta | + | + | 0.51 |
| PRCP | Lysosomal Pro-X carboxypeptidase (Fragment) | - | + |  |
| SERPINF2 | Alpha-2-antiplasmin (Fragment) | - | + |  |
| ACTBL2 | Beta-actin-like protein 2 | - | + |  |
| A2M | Alpha-2-macroglobulin | - | + |  |
| MLEC | Malectin (Fragment) | - | + |  |
| MYH9 | Myosin-9 | - | + |  |
| RGS13 | Regulator of G-protein signaling 13 | - | + |  |
| KCNIP4 | Isoform 3 of Kv channel-interacting protein 4 | - | + |  |
| PSMB1 | Proteasome subunit beta type-1 | - | + |  |
| DRP2 | Isoform 2 of Dystrophin-related protein 2 | - | + |  |
| EXD3 | Exonuclease mut-7 homolog, isoform 5 | - | + |  |
| KLHL38 | Kelch-like protein 38 | - | + |  |
| ATP5B | ATP synthase subunit beta (Fragment) | - | + |  |
| CNR1 | Isoform 2 of Cannabinoid receptor 1 | - | + |  |
| KIAA2012 | Uncharacterized protein KIAA2012 | - | + |  |
| HSPA1A | Isoform 2 of Heat shock 70 kDa protein 1A | - | + |  |
| SBSN | Suprabasin | - | + |  |
| MYOZ2 | Myozenin-2 | - | + |  |
| CCT5 | T-complex protein 1 subunit epsilon | - | + |  |
| NSD1 | Isoform 2 of Histone-lysine N-methyltransferase, H3 | - | + |  |
| DDX21 | Isoform 2 of Nucleolar RNA helicase 2 | - | + |  |
| S100A16 | Protein S100-A16 | + | + | 22.05 |
| MSI2 | RNA-binding protein Musashi homolog 2 (Fragment) | + | + | 13.07 |
| APOE | Apolipoprotein E | + | + | 11.37 |
| CAPNS1 | Calpain small subunit 1 (Fragment) | + | + | 5.92 |
| HSPA9 | Stress-70 protein, mitochondrial | + | + | 4.83 |
| HNRNPU | Isoform Short of Heterogeneous nuclear ribonucleoprotein U | + | + | 4.33 |
| DSP | Desmoplakin | + | + | 2.69 |
| C3 | Complement C3 | + | + | 2.26 |
| APOA1 | Apolipoprotein A-I | + | + | 2.19 |
| SERPINC1 | Antithrombin-III | + | + | 2.15 |
| PZP | Pregnancy zone protein | + | + | 2.08 |
| ALB | Serum albumin | + | + | 2.04 |
| TUBB | Tubulin beta chain | + | + | 1.95 |
| C4A | Complement C4-A | + | + | 1.89 |
| HRNR | Hornerin | + | + | 1.8 |
| KCTD3 | Isoform 2 of BTB/POZ domain-containing protein KCTD3 | + | + | 1.71 |
| ATP5A1 | Isoform 2 of ATP synthase subunit alpha, mitochondrial | + | + | 1.67 |
| a SGC-7901 cells infected with negative control lentivirus. | |  |  |  |
| b SGC-7901 cells infected with lentivirus overexpressing S100A16. | |  |  |  |
